# Gene abundance linked to climate zone: Parallel evolution of gene content along elevation gradients in lichenized fungi

**DOI:** 10.3389/fmicb.2023.1097787

**Published:** 2023-03-22

**Authors:** Dominik Merges, Francesco Dal Grande, Henrique Valim, Garima Singh, Imke Schmitt

**Affiliations:** ^1^Senckenberg Biodiversity and Climate Research Centre, Frankfurt am Main, Germany; ^2^LOEWE Centre for Translational Biodiversity Genomics (LOEWE-TBG), Frankfurt am Main, Germany; ^3^Department of Forest Mycology and Plant Pathology, Swedish University of Agricultural Sciences, Uppsala, Sweden; ^4^Department of Biology, University of Padova, Padua, Italy; ^5^National Biodiversity Future Center (NBFC), Palermo, Italy; ^6^Goethe University Frankfurt, Institute of Ecology, Evolution and Diversity, Frankfurt am Main, Germany

**Keywords:** environmental change, gene presences/absence, gene loss/gain, metagenomics, convergent evolution, population genomics, PacBio, protein kinase

## Abstract

**Introduction:**

Intraspecific genomic variability affects a species’ adaptive potential toward climatic conditions. Variation in gene content across populations and environments may point at genomic adaptations to specific environments. The lichen symbiosis, a stable association of fungal and photobiont partners, offers an excellent system to study environmentally driven gene content variation. Many of these species have remarkable environmental tolerances, and often form populations across different climate zones. Here, we combine comparative and population genomics to assess the presence and absence of genes in high and low elevation genomes of two lichenized fungi of the genus *Umbilicaria*.

**Methods:**

The two species have non-overlapping ranges, but occupy similar climatic niches in North America (*U. phaea*) and Europe (*U. pustulata*): high elevation populations are located in the cold temperate zone and low elevation populations in the Mediterranean zone. We assessed gene content variation along replicated elevation gradients in each of the two species, based on a total of 2050 individuals across 26 populations. Specifically, we assessed shared orthologs across species within the same climate zone, and tracked, which genes increase or decrease in abundance within populations along elevation.

**Results:**

In total, we found 16 orthogroups with shared orthologous genes in genomes at low elevation and 13 at high elevation. Coverage analysis revealed one ortholog that is exclusive to genomes at low elevation. Conserved domain search revealed domains common to the protein kinase superfamily. We traced the discovered ortholog in populations along five replicated elevation gradients on both continents and found that the number of this protein kinase gene linearly declined in abundance with increasing elevation, and was absent in the highest populations.

**Discussion:**

We consider the parallel loss of an ortholog in two species and in two geographic settings a rare find, and a step forward in understanding the genomic underpinnings of climatic tolerances in lichenized fungi. In addition, the tracking of gene content variation provides a widely applicable framework for retrieving biogeographical determinants of gene presence/absence patterns. Our work provides insights into gene content variation of lichenized fungi in relation to climatic gradients, suggesting a new research direction with implications for understanding evolutionary trajectories of complex symbioses in relation to climatic change.

## Introduction

Intraspecific genomic variability substantially affects a species’ adaptive potential to ecological interactions and climatic conditions (Hirsch et al., 2014; Badet et al., 2020; Resl et al., 2022). Variation in genomic content at the gene-level, i.e., presence/absences of genes, within species are regularly found in bacteria, and are often associated with adaptations to specific environments and antibiotic resistances (Masignani et al., 2005; Heuer et al., 2011). Recent studies suggest that eukaryotic species, like bacterial ones, can have intraspecific variation in genomic content at the gene-level (Hirsch et al., 2014; Badet et al., 2020; Gerdol et al., 2020). To this date, it is largely unknown how relevant such variation in gene content is for eukaryotes, and if it is ecologically important for adaptations to specific environments. Intraspecific variation in gene content contributes to the formation of genetically diverse populations, and therefore its characterization is vital to understand the mechanisms of adaptation to specific environments (Plissonneau et al., 2018; Drott et al., 2021; Merges et al., 2022).

The lichen symbiosis, a stable association of mainly fungal and photobiont partners as well as an associated microbiome, lends itself to the study of environmentally-driven gene content, because many species have remarkable environmental tolerances and maintain populations in different climate zones (Kappen, 2000; Werth and Sork, 2014; Singh et al., 2017; Grimm et al., 2021; Jung et al., 2021; Tanunchai et al., 2022). Previous studies showed that environmental differentiation in lichens can be found at the level of single nucleotide polymorphisms (SNPs), which often significantly correlate with differences in geography and ecology and may thus be involved in environmental specialization (Peksa and Skaloud, 2011; Castillo et al., 2012; Hodkinson et al., 2012). Population genomic analyses based on SNPs suggest the presence of genome-wide differentiation between populations in different climate zones (Dal Grande et al., 2017; Rolshausen et al., 2022). To date, the only study which assesses variation in gene content associated with environmental adaptation in lichens is limited to a single species: Singh et al. (2021) show that some gene clusters associated with natural product biosynthesis in *Umbilicaria pustulata* have elevation-specific distributions. However, we currently lack information on the extent of parallel evolution in populations of different species that have independently evolved under similar environmental conditions. We do not know, e.g., (1) whether different species of lichen-forming fungi maintain homologous population-specific genes along comparable environmental gradients and (2) whether intraspecific variation in gene content (e.g., abundance patterns of genes) has independently converged on the same altitudinal patterns in different species, and can thus be linked to environmental preferences of lichens.

Modern evolutionary approaches leverage DNA sequencing to infer ecological and evolutionary processes that occur at the population level. Here we combine comparative genomics and population genomics to assess the presence/absence of genes in high elevation and low elevation genomes of two lichenized fungi of the genus *Umbilicaria* (*Umbilicaria phaea* and *U. pustulata*). The two species have evolved on different continents under similar climatic selective pressures: *U. pustulata* in Europe and *U. phaea* in North America each occupy the cold temperate as well as the Mediterranean climate zone. We tracked gene content variation along replicated elevation gradients in both species based on a total of 2050 individuals in 26 populations. Specifically, we addressed the following research questions: (a) Which genes are linked to environmental conditions at high elevation (cold temperate climate) and low elevation (Mediterranean climate) across species? To address this question, we assessed which genes are exclusive to climate zones. (b) Do abundances of genes specific to a climate zone co-vary with elevation in populations of *U. phaea* and *U. pustulata*? To address the second question, we assessed which genes increase or decline in abundance with increasing elevation.

## Materials and methods

### Study site and sample collection

We sampled 15 *U. pustulata* populations along three elevational gradients in Spain and Italy and 11 *U. phaea* populations along two elevational gradients in California, United States (Merges et al., 2021; Singh et al., 2022). Our choice of gradients was based on the high abundance of *U. pustulata* and *U. phaea* in these respective areas, and because the gradients span two contrasting bioclimates, the Mediterranean and the cold temperate zones. A population of lichens is here defined as a group of individuals collected on rocks within an area of approximately 10 × 10 m. Detailed sampling procedures are described in Dal Grande et al. (2017) and Rolshausen et al. (2020). The altitudinal spacing of populations along the gradients can be seen in Supplementary Table S1. The studied *Umbilicaria* species entail foliose, monophyllous lichens, which are attached to rock surfaces with a central holdfast. This growth form facilitates the recognition of individuals. Details of the sampling of European material are described in Singh et al. (2022) and the details of sampling of the North American material in Dal Grande et al. (2017) and Merges et al. (2021). Briefly, two of the European gradients are located in Central Spain, Sierra de Gredos (40.2028, −5.2334 and 39.9946, −4.8679) and one on the island of Sardinia (40.7577, 9.0794). We collected fragments of 100 individuals each, at Mount Limbara (Sardinia, Italy; 6 populations), Sierra de Gredos (Sistema Central, Spain; 6 populations) and Talavera-Puerto de Pico (Sistema Central, Spain; 3 populations), as described in Dal Grande et al. (2017). The Californian gradients are spatially separated by approx. 700 km. We collected fragments of 50 individuals each, at four populations along the Sierra Nevada gradient (38.084, −120.484) and at seven population along the Mt. Jacinto gradient (33.435, −116.484). Schemes indicating the geographic location and sites of the sample collection are given in Supplementary Table S1. We additionally collected four whole lichen thalli, one low-altitude individual from the Sierra Nevada population and one from the high-altitude population, as well as a low-altitude and a high-altitude individual from populations of the Sierra des Gredos gradient for the reconstruction of reference genomes using PacBio Sequel II data (Merges et al., 2021; Singh et al., 2022; Supplementary Table S2).

### DNA extraction for population pooled sequencing

Genomic DNA was extracted separately from each fragment from all populations using a cetyltrimethylammonium bromide-based (CTAB) method (Cubero and Crespo, 2002; Dal Grande et al., 2017). Further, we created a pooled sample for each population containing equal amounts of DNA from each sample and Novogene Co., Ltd. (Cambridge, United Kingdom) performed the library preparation (200–300 bp insert size; Dal Grande et al., 2017). Libraries were sequenced on an Illumina HiSeq2000 with 150 bp paired-end chemistry at ~90× coverage per population (i.e., Pool-seq; Dal Grande et al., 2017).

### DNA extraction for genomic sequencing

Genomic DNA for genome sequencing was extracted from dry thallus material of two samples of the same species (i.e., *U. phaea* or *U. pustulata*) collected in different climatic zones (i.e., low elevation/Mediterranean climate zone and high elevation/temperate climate zone). Lichen thalli were thoroughly washed with sterile water and checked under the stereomicroscope for the presence of possible contamination or other lichen thalli. DNA was extracted from all of the samples using CTAB-based method (Mayjonade et al., 2016) as presented in Merges et al. (2021).

### PacBio library preparation and sequencing

For PacBio single-molecule real-time (SMRT) sequencing, SMRTbell libraries were constructed according to the manufacturer’s instructions of the SMRTbell Express Prep kit v2.0 following the Low DNA Input Protocol (Pacific Biosciences, Menlo Park, CA, United States). Total input DNA was approximately 140 and 800 ng, respectively. Ligation with T-overhang SMRTbell adapters was performed at 20°C overnight. Following ligation, the SMRTbell library was purified with an AMPure PB bead clean up step with 0.45X volume of AMPure PB beads. Subsequently a size-selection step with AMPure PB Beads was performed to remove short SMRTbell templates <3 kb. For this purpose, the AMPure PB beads stock solution was diluted with elution buffer (40% volume/volume) and then added to the DNA sample with 2.2X volume. The size and concentration of the final libraries were assessed using the TapeStation (Agilent Technologies) and the Qubit Fluorometer with Qubit dsDNA HS reagents Assay kit (Thermo Fisher Scientific, Waltham, MA, United States). Sequencing primer v4 and Sequel^®^ II Polymerase 2.0 were annealed and bound, respectively, to each SMRTbell library. SMRT sequencing was performed on the Sequel System II with Sequel II Sequencing Kit 2.0 in “continuous long read” (i.e., CLR) mode, 30 h movie time with no pre-extension and Software SMRTLINK 8.0 (Pacific Biosciences of California, 2022). One SMRT Cell was run for each sample. A SMRT cell contains millions of wells called zero-mode waveguides (ZMWs; Pacific Biosciences of California, 2022). Within each ZMWs single molecules of DNA are immobilized and as the polymerase incorporates each nucleotide, light is emitted, and nucleotide incorporation is measured in real time (Pacific Biosciences of California, 2022).

### *De novo* assembly of PacBio metagenomic sequence reads

We largely followed the pipeline described in Merges et al. (2021). In summary, we generated HiFi reads from the PacBio Sequel II run using the PacBio tool CCS v5.0.0 with default parameters, i.e., --min-passes 3, remove subreads with lengths <50% or > 200% of the median subread length, −-max-insertion-size to 30 bp (Pacific Biosciences of California, 2022).^1^ Metagenomic sequence reads were assembled into contigs using the long-read based assembler metaFlye v2.7 (Kolmogorov et al., 2019). The assembled contigs were scaffolded with LRScaf v1.1.12 (Qin et al., 2019).^2^ To retrieve the mycobiont genome, the received scaffolds were taxonomically binned *via* blastx using DIAMOND (−-more-sensitive --frameshift 15 –range-culling) on a custom database (Singh et al., 2022) and the Metagenome Analyzer MEGAN6 Community Edition pipeline (Huson et al., 2007; Buchfink et al., 2014). The completeness of the genomes represented by the binned Ascomycota scaffolds was estimated using Benchmarking Universal Single-Copy Orthologs (BUSCO) analysis in BUSCO v4 using the Ascomycota dataset (Simão et al., 2015).

### Pool-seq data processing

We filtered the pool-seq data for reads shorter than 80 bp, reads with N’s, and reads with average base quality scores less than 26 along with their pairs, and discarded them. We mapped the trimmed paired-end reads of each pool to the database of the identified genes using bowtie2 v2.4.1 (Langmead and Salzberg, 2012), using the flags: --very-sensitive-local, −-no-mixed, −-no-unal, −-no-discordant.

### Gene prediction and genome annotation

Functional annotation of genomes, including genes and proteins (antiSMASH; antibiotics and SM Analysis Shell, v5.0) was performed with scripts implemented in the funannotate pipeline (Blin et al., 2017; Palmer and Stajich, 2019). First, the genomes were masked for repetitive elements, and then the gene prediction was performed using BUSCO2 to train Augustus and self-training GeneMark-ES (Borodovsky and Lomsadze, 2011; Simão et al., 2015). Functional annotation was done with InterProScan (Quevillon et al., 2005), egg-NOG-mapper (Huerta-Cepas et al., 2017, 2019), and evolutionarily-informed expectations of gene content of near-universal single-copy orthologs using BUSCO v 5.1.2 with included Ascomycota dataset (Simão et al., 2015). Secreted proteins were predicted using SignalP (Armenteros et al., 2019) as implemented in the funannotate “annotate” command. Proteins where further characterized by NCBI conserved domain search (Lu et al., 2020).^3^

### Assessing gene content variation in the assembled fungal genomes

To identify presence/absences patterns of genes, we identified orthologs using orthoFinder (Emms and Kelly, 2015, 2019). OrthoFinder provides the most accurate ortholog inference method on the Quest for Orthologs benchmark test (Emms and Kelly, 2015, 2019). In orthoFinder (v.2.5.4), we assigned all genes to orthogroups using protein homology and constructed a pangenome of all four complete genomes (Badet et al., 2020). Shared orthologs (i.e., members of the some orthogroup) of low elevation (warm adapted) and high elevational (cold adapted) genomes were extracted using R v3.6.1 (R Core Team, 2019).

### Validating presence/absence of genes at population level

To validate population-level gene presence or absence, we estimated the abundance of each ortholog in the low elevation (warm adapted) and high elevation (cold adapted) population based on the median coverage of pool-seq reads associated to each ortholog contig. Specifically, we used samtools (v1.15) depth to estimate the coverage of each basepair within the contig (Danecek et al., 2021). We assessed and visualized the data in R v3.6.1 (R Core Team, 2019).

### Gene distribution across *Umbilicaria* populations

Bowtie2 (v2.2.2) was used to map pool-seq reads to all ortholog contigs (using default settings). The number of mapped reads was counted per sample and normalized by dividing the number of mapped reads by the total read number of the respective sample to account for differences in sequencing depth. We modeled gene abundance (i.e., normalized read count) as a function of elevation using linear models. Linear models were fitted and plotted in R v3.6.1 (R Core Team, 2019).

## Results

### HiFi metagenomic sequencing reads of mycobiont

We reconstructed metagenomic sequences from a low-elevation and a high-elevation specimen of *U. pustulata* and *U. phaea*. Sequence output and quality for *U. pustulata* were summarized in Singh et al. (2022), for *U. phaea* in Merges et al. (2021) and in Supplementary Table S2.

### Altitude-specific genes in the *de novo* assembled genomes

**Figure 1:**
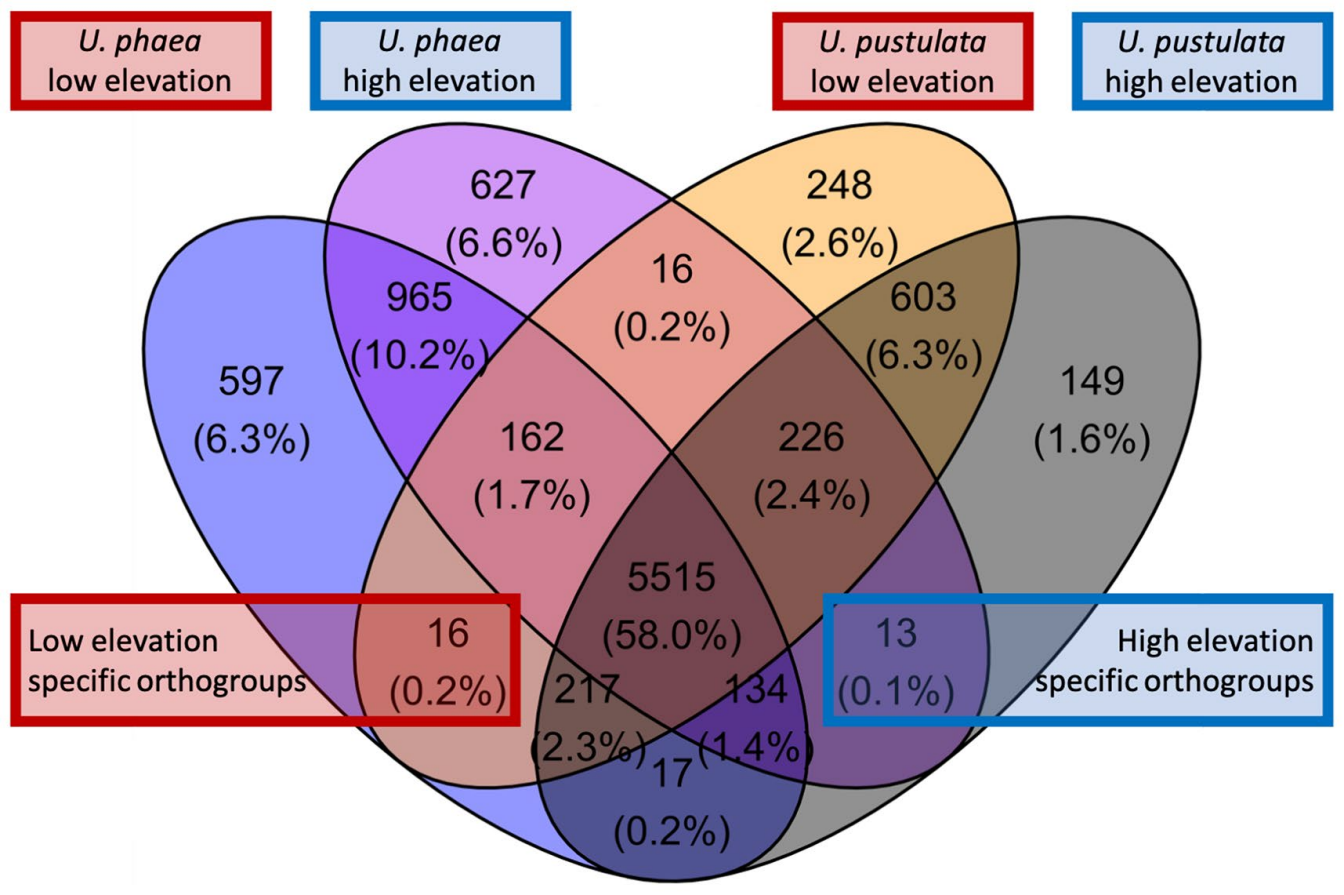
Venn diagram displaying orthogroups of *U. phaea* and *U. pustulata*. Red box highlights orthologs of the warm adapted (low elevation) *U. phaea* and *U*. *pustulata* genomes and the blue box of the cold adapted (high elevation) genomes.

We screened the de-novo assembled genomes of the low-and high-altitude samples for altitude-specific genes (Figure 1). Orthofinder revealed 16 orthogroups with shared orthologous genes (0.2% of the total orthogroups) in low-elevation genomes and 13 in high-elevational genomes (0.1% of the total orthogroups).

### Presence/absence of genes at population level

**Figure 2:**
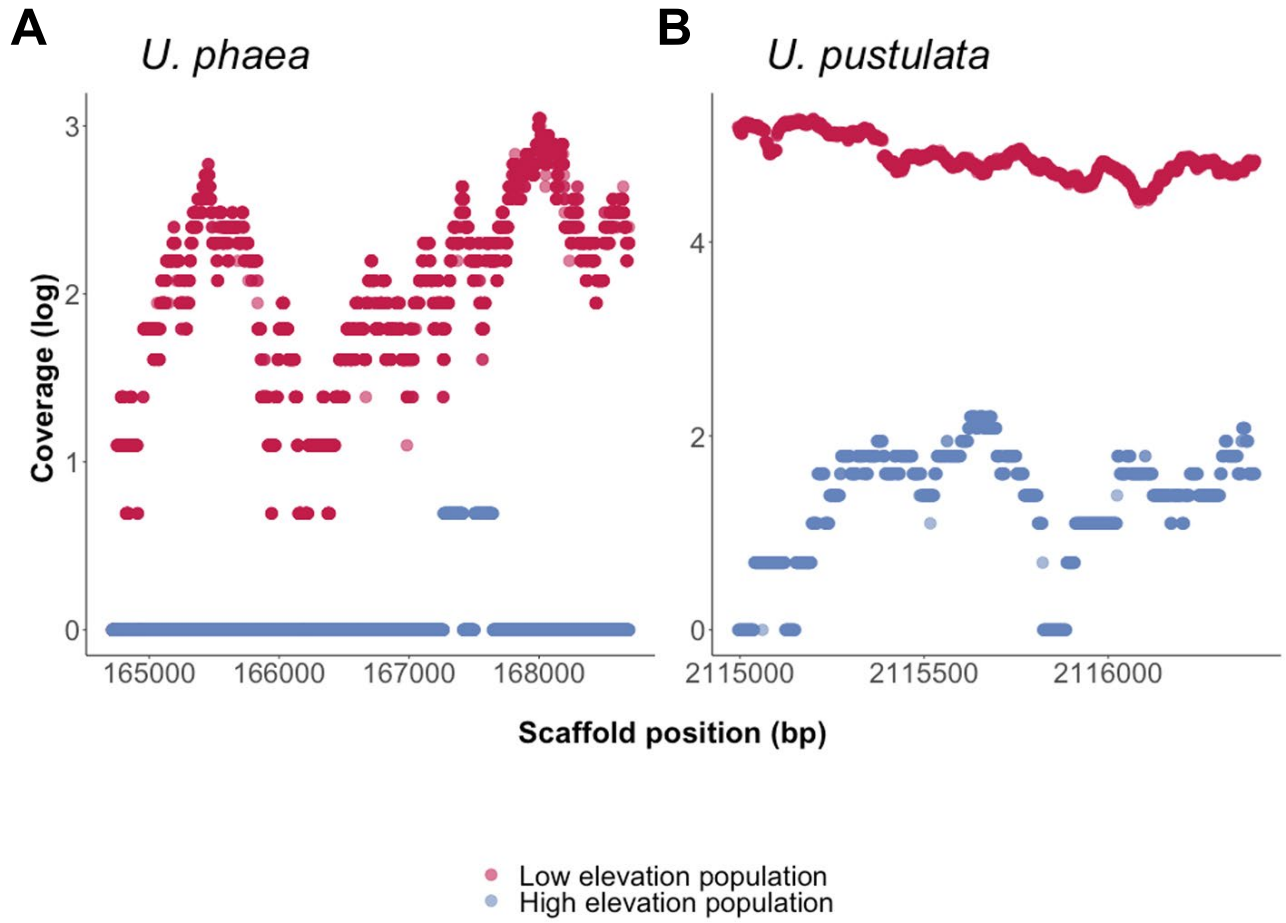
Presence/absences of orthologs in four *de novo* sequenced genomes of *U. phaea* and *U. pustulata* were verified by assessing the scaffold coverage in the warm adapted (low elevation) and the cold adapted population (high elevation) respectively. **(A)** Coverage of ortholog in *U. phaea*: High coverage in warm adapted (low elevation) population and no coverage in high elevational population. **(B)** Coverage of ortholog in *U. pustulata*: High coverage in warm adapted (low elevation) population.

To verify the presence/absence of detected orthologs, the coverage of each ortholog was calculated for the respective population at low and high elevation. The coverage analysis revealed one ortholog present in the genomes at low elevation to be consistently absent in populations at high elevation (Figure 2). The amino acid sequences of the ortholog could not be functionally annotated using the funannotate pipeline and was classified as “hypothetical protein.” NCBI’s conserved domain search revealed an alignment with the catalytic domain of protein kinase superfamily member PKc cd00180 (Position-specific scoring matrix (PSSM) accession cl214531, NCBI *Conserved Domain Database*) as well as seven Tetratricopeptide repeats (Tetratricopeptide-like helical domain superfamily, InterPro entry IPR011990), indicating putative protein binding surfaces (Supplementary Figure S1).

### Gene abundance distributions along gradients

**Figure 3:**
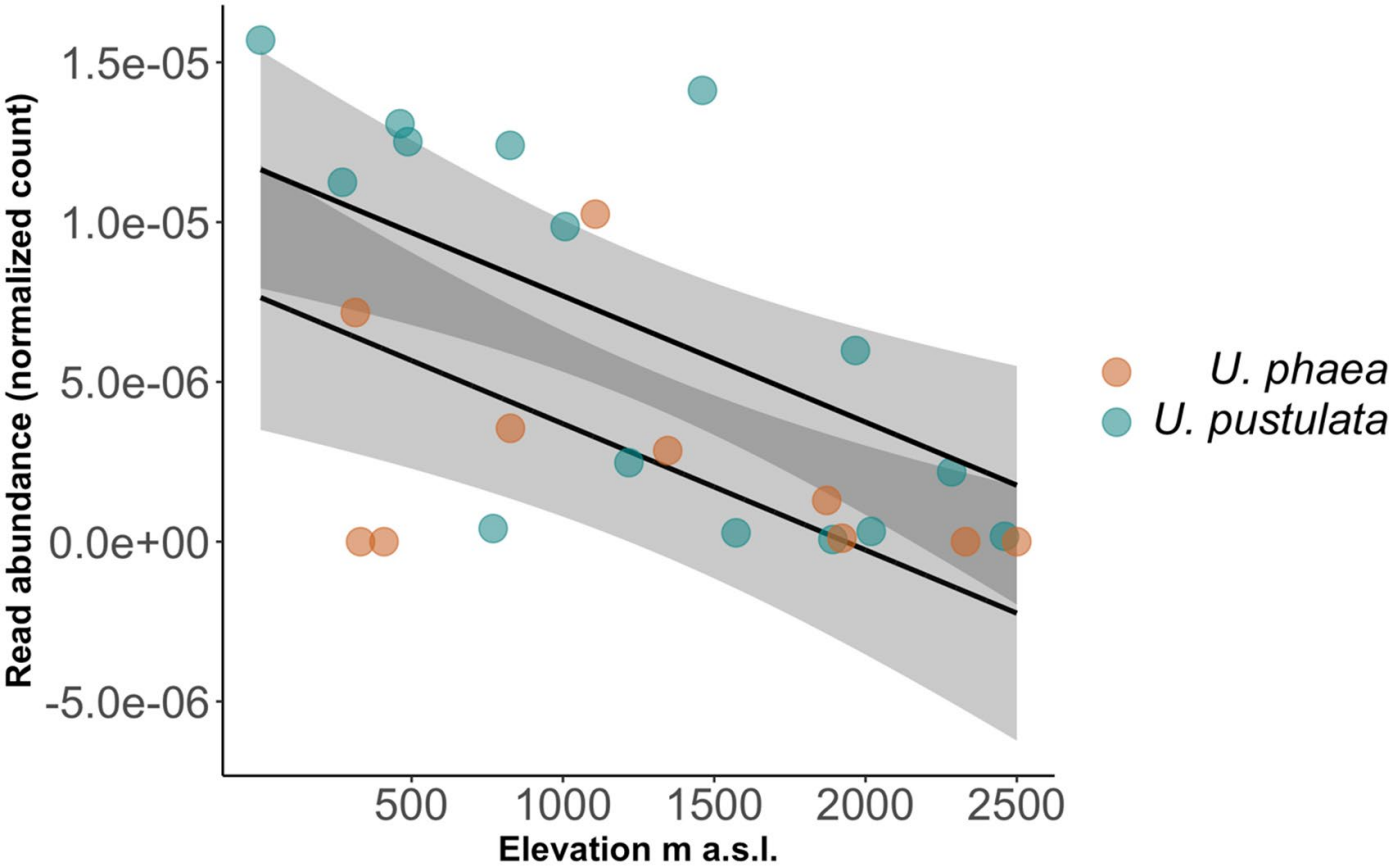
Read abundance of identified ortholog decreases significantly with increasing elevation across *U. phaea* (brown circles, lower regression line) and *U. pustulata* (blue circles, upper regression line) populations.

The normalized read number of the identified orthologs, annotated as members of the Protein Kinases superfamily, showed a decline with increasing elevation across all populations in both species (value of *p* = 0.00373, Figure 3).

## Discussion

Although adaptations to environmental gradients may lead to variation in gene content, assessments of gene presence/absence patterns across populations and species of lichenized fungi are still missing. Here we assess signatures of parallel evolution at the level of gene presence and absence in lichenized fungi of the genus *Umbilicaria*, and trace the discovered genes in lichen populations along five replicated elevation gradients across two continents. While our whole genome comparison based on four *de novo* sequenced specimen (two per species) suggested up to 29 elevation-specific orthogroups (i.e., 16 low elevation-specific and 13 high elevation-specific orthogroups, Figure 1), the population-level verification approach showed only one gene, putatively encoding a protein kinase (PK), which linearly declined in abundance with increasing elevation, and was truly absent in the highest population. This suggests either high strain-specificity of certain genes, or high false positive recovery of gene presence/absence patterns when relying on comparative genomics approaches based on only a few individuals. Thereby extrapolating the significance of gene content variation assessed only with comparative genomics approaches on a few individuals can be potentially misleading when interpreting the evolutionary significance of the variation at population level. Regarding the PK gene consistently absent in high elevation genomes and populations, we found that the discovered gene declines linearly across all populations, suggesting an evolutionary benefit only at lower altitudes. Alternatively, the loss of the gene at higher elevations might benefit individuals in cold climates. To our knowledge, we report for the first time parallel gene presence and absence patterns correlating with climatic niches in different species of lichenized fungi. However, it remains to be analyzed, if the identified molecular trait is associated to a particular phenotype that can be associated with an adaptive function.

In bacteria variation in gene content is assumed to be driven by selection for environmental conditions that are relatively rare across the entire range of a species (Qi et al., 2017). Recent evidence suggests that specific populations of lichenized fungi may contain unique biosynthetic gene clusters (Singh et al., 2021), and our current findings show that also other genes can be elevation-specific. The gradual gene loss across populations with increasing elevation may suggests a decline of selective benefit and may indicate that certain variations in gene content could be of functional importance for local adaptation and climatic tolerances in lichenized fungi. The conserved domain search revealed a catalytic domain of a PK, a common eukaryotic protein superfamily. PKs selectively modify other proteins by phosphorylation, changing their enzymatic activity, cellular location and association with other proteins (Cheng et al., 2002; Hanks, 2003; Asano et al., 2005; Heinisch and Rodicio, 2018). Within a genome, PKs are encoded by a large multigene family with genes being distributed among multiple chromosomes. Putatively, the high number of PK genes has arisen by genome segmental duplication events (Asano et al., 2005; Heinisch and Rodicio, 2018). In our study, the presence/absence of a single PK gene may suggest a climate-specific ancestral genome segmental duplication event. Across the tree of life, e.g., in bacteria (Christ and Chin, 2008), fungi (James et al., 2008) and plants (DeBolt, 2010; Prunier et al., 2019), organisms subjected to selection under high temperatures show higher probability for genome segmental duplications (Christ and Chin, 2008; James et al., 2008; DeBolt, 2010; Kondrashov, 2012). For example, adaptation to heat stress through gene duplication of stress-related genes has been shown in *Escherichia coli* (Riehle et al., 2001; Kondrashov, 2012), where an upregulation through gene duplication of genes may play a role in adaptation (Kondrashov, 2012; Kuzmin et al., 2022). However, due to the scarcity of functional annotation of non-model organisms and the resulting lack of in-depth functional annotation of the gene in question, the mechanisms generating such population level diversification and the underlying molecular mechanisms behind such adaptations are yet to be understood.

While environmental adaptations are commonly highly polygenic (Rivas et al., 2018; Barghi et al., 2019; Hartke et al., 2021; Pfenninger et al., 2021), there is increasing evidence of the effect of single gene content variation (Liu et al., 2021). For example, as has been recently shown in agave, where a single gene encoding a phosphoenolpyruvate carboxylase enhances the plant’s climate resilience (Liu et al., 2021). Not only the gain of genes, but also the loss of genes has been associated with adaptive traits, such as the evolution of particular diets in bats (Blumer et al., 2022). Therefore, we consider the parallel loss of a homologous gene in two species and two geographic settings a rare find, and a step forward in understanding the genomic underpinnings of climatic tolerances in lichenized fungi. Future research should address the functional importance of the gene present at low altitude in the Mediterranean climate zone, and specifically explore the effects of variation in gene abundances across populations. Additionally, future research should consider using heterologous expression approaches to reveal whether the gene presence could induce tolerances to warm conditions.

## Conclusion

Our study demonstrates how comparative genomics in combination with population genomic data can reveal patterns of gene content variation across climatic gradients. In addition, the tracking of gene content variation across populations provides a widely applicable framework for retrieving meaningful biogeographical determinants of gene presence/absence patterns. We contribute to understanding convergence and parallel evolution at the genomic level, by providing insights into gene content variation of lichenized fungi in relation to climatic gradients. This suggests a promising new research direction with implications for understanding evolutionary trajectories in relation to climatic change.

## Data availability statement

Raw sequence reads were deposited in the National Centre for Biotechnology Information (NCBI) Sequence Read Archive under the BioProject accession numbers PRJNA693984 and PRJNA820300.

## Author contributions

DM and IS conceived the ideas and wrote the manuscript. IS and FD collected the data. DM, GS, and HV performed genome assembly and annotations. DM analyzed the data. FD provided analytical guidance. All authors contributed to the various drafts and gave final approval for publication.

## Funding

This research has been partly funded by the Hesse’s “state initiative for the development of scientific and economic excellence” (LOEWE initiative) through the LOEWE Center for Translational Biodiversity Genomics (TBG).

## Conflict of interest

The authors declare that the research was conducted in the absence of any commercial or financial relationships that could be construed as a potential conflict of interest.

## Publisher’s note

All claims expressed in this article are solely those of the authors and do not necessarily represent those of their affiliated organizations, or those of the publisher, the editors and the reviewers. Any product that may be evaluated in this article, or claim that may be made by its manufacturer, is not guaranteed or endorsed by the publisher.

## Footnotes

^1^ https://ccs.how

^2^ https://github.com/shingocat/lrscaf

^3^ https://www.ncbi.nlm.nih.gov/Structure/cdd/wrpsb.cgi
