# Supplementary material for: Gene abundance linked to climate zone: Parallel evolution of gene content along elevation gradients in lichenized fungi
Source: Front Microbiol. 2023 Mar 22;14:1097787. doi: 10.3389/fmicb.2023.1097787 (PMC10073550; doi:10.3389/fmicb.2023.1097787)
Supplement: Supplementary file 1 [file Data_Sheet_1.docx]

Gene abundance linked to climate zone: parallel evolution of gene content along elevation gradients in lichenized fungi

Dominik Merges^1,2,3*^, Francesco Dal Grande^1,2,4,6^, Henrique Valim^1,2^, Garima Singh^1,2,4^, Imke Schmitt ^1,2,^

Supplement

Table S1: Population IDs, coordinates and elevations U. phaea and U. pustulata populations along five elevational gradients.

| **Gradient** | **Site** | **Elevation m a.s.l.** | **Latitude** | **Longitude** |
| --- | --- | --- | --- | --- |
| Mount San Jacinto | J1 | 646 | 33.435 | -116.484 |
|  | J2 | 714 | 33.406 | -116.503 |
|  | J3 | 1079 | 33.421 | -116.461 |
|  | J4 | 1324 | 33.422 | -116.441 |
|  | J5 | 1992 | 33.456 | -116.411 |
|  | J6 | 2393 | 33.463 | -116.404 |
|  | J7 | 2541 | 33.484 | -116.382 |
| Sierra Nevada | N1 | 631 | 38.084 | -120.484 |
|  | N2 | 1014 | 38.199 | -120.364 |
|  | N3 | 1533 | 38.288 | -120.3 |
|  | N4 | 2036 | 38.398 | -120.166 |
| Mount Limbara | L1 | 117 | 40.758 | 9.079 |
|  | L2 | 310 | 40.778 | 9.055 |
|  | L3 | 428 | 40.798 | 9.064 |
|  | L4 | 588 | 40.85 | 9.112 |
|  | L5 | 643 | 40.86 | 9.127 |
|  | L6 | 844 | 40.857 | 9.134 |
|  | L7 | 1117 | 40.857 | 9.164 |
|  | L8 | 1307 | 40.852 | 9.173 |
| Sierra de Gredos | G1 | 706 | 40.203 | -5.233 |
|  | G2 | 887 | 40.207 | -5.233 |
|  | G3 | 1082 | 40.212 | -5.234 |
|  | G4 | 1258 | 40.218 | -5.233 |
|  | G5 | 1480 | 40.225 | -5.238 |
|  | G6 | 1699 | 40.232 | -5.239 |
| Talavera-Puerto de Pico | P1 | 477 | 39.9946 | −4.8679 |
|  | P2 | 859 | 40.2899 | −4.9927 |
|  | P3 | 1417 | 40.323 | −5.0173 |

Table S2: Genome quality and annotation statistics

| **Taxon** | **Sample ID** | **CCS**  **HiFi yield (%)** | **No. of scaffolds** | **N50** | **Completeness**  **(%)** | **Assembly size**  **(Mb)** | **No.**  **of genes** | **No. of proteins** | **Genome accession** |
| --- | --- | --- | --- | --- | --- | --- | --- | --- | --- |
| *U. phaea 1* | TBG_1111 | 5.72 | 47 | 1.54 | 96.5 | 35.1 | 7,659 | 7,576 | SRX9925339 |
| *U. phaea 2* | TBG_1112 | 22.36 | 38 | 1.22 | 96.5 | 35.55 | 7,681 | 7,628 | SRX9925340 |
| *U. pustulata 1* | TBG_2333 | 33 | 26 | 2.62 | 97.3 | 37.7 | 9,569 | 9,503 | JALILU000000000 |
| *U. pustulata 2* | TBG_2345 | 32.26 | 31 | 2.36 | 96.8 | 35.7 | 8,790 | 8,740 | JALILV000000000 |


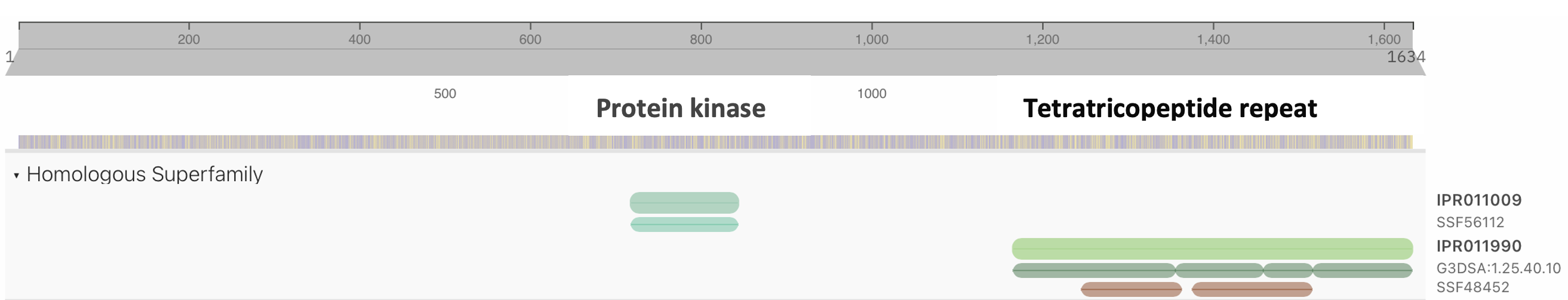


Figure S1: Protein features of the gene region: The amino acid sequence showing the position of the catalytic domain of Protein Kinases superfamily member PKc cd00180 (InterPro entry IPR011009) as well as the Tetratricopeptide repeats (Tetratricopeptide-like helical domain superfamily, InterPro entry IPR011990), indicating putative protein binding surfaces.
